# Peptide Lv augments intermediate-conductance calcium-dependent potassium channels (K_Ca_3.1) in endothelial cells to promote angiogenesis

**DOI:** 10.1371/journal.pone.0276744

**Published:** 2022-10-25

**Authors:** Dylan L. Pham, Autumn Niemi, Michael L. Ko, Gladys Y. P. Ko

**Affiliations:** 1 Department of Veterinary Integrative Biosciences, College of Veterinary Medicine and Biomedical Sciences, Texas A&M University, College Station, Texas, United States of America; 2 Department of Biology, Division of Natural and Physical Sciences, Blinn College, Bryan, Texas, United States of America; 3 Texas A&M Institute for Neuroscience, Texas A&M University, College Station, Texas, United States of America; University of Hull, UNITED KINGDOM

## Abstract

Peptide Lv is a small endogenous secretory peptide that is expressed in various tissues and conserved across different species. Patients with diabetic retinopathy, an ocular disease with pathological angiogenesis, have upregulated peptide Lv in their retinas. The pro-angiogenic activity of peptide Lv is in part through promoting vascular endothelial cell (EC) proliferation, migration, and sprouting, but its molecular mechanism is not completely understood. This study aimed to decipher how peptide Lv promotes EC-dependent angiogenesis by using patch-clamp electrophysiological recordings, Western immunoblotting, quantitative PCR, and cell proliferation assays in cultured ECs. Endothelial cells treated with peptide Lv became significantly hyperpolarized, an essential step for EC activation. Treatment with peptide Lv augmented the expression and current densities of the intermediate-conductance calcium-dependent potassium (K_Ca_3.1) channels that contribute to EC hyperpolarization but did not augment other potassium channels. Blocking K_Ca_3.1 attenuated peptide Lv-elicited EC proliferation. These results indicate that peptide Lv-stimulated increases of functional K_Ca_3.1 in ECs contributes to EC activation and EC-dependent angiogenesis.

## Introduction

Pathological angiogenesis/neovascularization manifests in various diseases including cancers [1], atherosclerosis [2], arthritis [3], and ocular diseases such as diabetic retinopathy (DR) and age-related macular degeneration [4–6]. Therapies targeting vascular endothelial growth factor (VEGF) or its receptors are widely used to combat these diseases by dampening neovascularization [7–9]. However, nearly 30% of patients do not respond or become resistant to anti-VEGF therapies [9–11]. Repetitive anti-VEGF injections are needed to block recurring neovascularization, which often leads to unwanted side effects [9–11]. One possible explanation for the resistance to anti-VEGF treatments and recurring neovascularization is the involvement of other angiogenic factors that are insensitive to anti-VEGF agents [9–11]. Thus, finding VEGF-independent pro-angiogenic factors and understanding their molecular mechanisms become clinically imperative for the development of new therapeutics against pathological neovascularization.

We discovered a small endogenous peptide (~40 amino acids), peptide Lv, that is upregulated in the retinas of patients with early proliferative diabetic retinopathy (DR) as well as diabetic animals [12–14]. The gene encoding peptide Lv is in the V-set and transmembrane domain containing 4 gene (*Vstm4*; human gene ID: 196740, a.a. 55–94; mouse gene ID: 320736, a.a. 55–103; [12]). Its amino acid sequence is highly conserved (>90%) among humans, mice, rats, and chickens [12]. The mRNA of peptide Lv is expressed in various organs including the eye, heart, brain, liver, spleen, and lung [12] and cell types including retinal neurons, vascular endothelial cells (ECs), and cardiomyocytes [12, 13]. Functionally, peptide Lv elicits concentration-dependent vasodilation in porcine coronary and retinal arterioles [14], similar to how VEGF acts as a vasodilator [15]. Peptide Lv is also pro-angiogenic since it promotes developmental and pathological angiogenesis *in vivo* [14]. During development, chicken embryos treated with peptide Lv (*in ovo*) or postnatal mice injected with peptide Lv intraocularly have significant growth of microvasculature [14]. Mouse eyes with oxygen-induced retinopathy (OIR) have upregulated peptide Lv [14], which is similar to the upregulation of VEGF in these eyes [7, 8, 16, 17]. Intraocular injections with an antibody against peptide Lv, anti-Lv, dampen pathological neovascularization in mouse eyes with OIR or laser-induced choroidal neovascularization [14]. Furthermore, mice with a genetic deletion of peptide Lv (peptide Lv^-/-^) have significantly higher OIR-induced vaso-obliteration and lower OIR-neovascularization compared to the littermate controls (peptide Lv^+/+^; [14]). These data provide evidence that peptide Lv is an angiogenic factor that is involved in pathological neovascularization.

Upon further investigation, peptide Lv elicits vascular EC proliferation, migration, and sprouting, which are three fundamental properties in angiogenesis [14]. One of the initial steps for EC-dependent vasodilation or angiogenesis is the membrane hyperpolarization of ECs [18–22], and opening EC potassium (K^+^) channels is required for EC hyperpolarization [18–20, 23–27]. Several K^+^ channels expressed in ECs can mediate the outflow of K^+^, including intermediate conductance calcium-dependent K^+^ channels (IKCa/KCNN4/K_Ca_3.1) [25, 26, 28], small conductance calcium-dependent K^+^ channels (sKCa/KCNN3/K_Ca_2.3) [25, 26, 28], and ATP-sensitive K^+^ channels (K_ATP_/Kir6.1) [19, 29–31].

Since it is likely that peptide Lv-elicited vasodilation and angiogenesis is in part through an EC-dependent process, we postulated that peptide Lv might elicit EC hyperpolarization through activating EC-expressed K^+^ channels. In this study, we combined patch-clamp electrophysiological recordings and molecular analyses to determine whether peptide Lv caused any changes in EC membrane potential, and which EC-expressed K^+^ channel(s) is involved and leads to peptide Lv-elicited angiogenesis.

## Materials and methods

This study (agents and experimental activities) was approved by the Institutional Biosafety Committee (IBC) of Texas A&M University (IBC Permit: IBC 2020–104).

### Chemicals

Peptide Lv was custom-made by Peptide 2.0 Inc (Chantilly, VA, USA). The murine amino acid sequence used to make peptide Lv is DSLLAVRWFFAPDGSQEALMVKMTKLRIIQYYGNFSRTANQQRLRLLEE [12, 13]. Peptide Lv tested negative for endotoxin. Other inhibitors and chemicals used in this study were: TRAM-34 (K_Ca_3.1 inhibitor; #AAJ60019-MB, Thermo Fisher Scientific, Waltham, MA, USA), DMH4 (VEGFR2 inhibitor; #4471, Tocris, Minneapolis, MN, USA), β-escin (#E1378, Sigma-Aldrich, St. Louis, MO, USA). and VEGF (#ab9571, Abcam, Waltham, MA, USA).

### Cell cultures

Human umbilical vein endothelial cells (HUVECs; #200-05n, Cell Applications Inc, San Diego, CA, USA) and human retinal endothelial cells (HRECs; #ACBRI 181, Cell Systems, Kirkland, WA, USA) were cultured in EGM^™^ -2 MV Microvascular Endothelial Cell Growth Medium (EGM; #CC-3202, Lonza, Walkersville, MD, USA) at 37ºC and 5% CO_2_. For immunoblot and qPCR experiments, ECs were seeded onto 60 mm culture plates and grown to 100% confluency then treated with peptide Lv (500 ng/ml) or phosphate-buffered saline (PBS; vehicle control) for 4 hours. For patch-clamp experiments, ECs were seeded onto 12 mm acid-washed glass coverslips and placed in an incubator for 48 hours to allow cells to adhere. Cultures were then treated with peptide Lv (500 ng/ml) or PBS for 2, 3, and 4 hours prior to recordings. For cell proliferation assays, HUVECs were seeded onto 96-well plates in EGM and allowed to adhere overnight. Peptide Lv (500 ng/ml), VEGF (5 ng/ml), DMH4 (5 μM), and TRAM-34 (10 μM) were added to cells and continuously incubated for another 48 hours prior to proliferation assays.

### Patch-clamp electrophysiology

Whole-cell patch-clamp recordings on cultured HUVECs were carried out using β-escin-based perforated patches [32–34]. The methods and parameters for recording endothelial membrane potentials (current-clamp) and the K_Ca_3.1 current (voltage-clamp) are based on previous publications [35–37]. The external solution was (in mM): 160 NaCl, 4.5 KCl, 1 MgCl_2_, 2 CaCl_2_, 10 HEPES, and 1 glucose at pH 7.5 adjusted with NaOH. The pipette solution was (in mM): 120 KCl, 1.75 MgCl_2_, 1 Na_2_ATP, 10 EGTA, 4.1 CaCl_2_, and 10 HEPES at pH 7.2 adjusted with KOH. The free calcium concentration in the pipette solution was calculated to be 100 nM using an online calcium chelator calculator [38]. β-escin was freshly prepared as a 35 mM stock solution in water, kept on ice, and then added to the pipette solution to yield a final concentration of 35 μM. Before recordings, cells were first treated with peptide Lv (500 ng/ml) or PBS (vehicle; as controls) and maintained in the incubator for 0, 2, 3, or 4 hours. There was no statistical difference in amplitudes (either membrane potentials or currents) among the controls recorded, so their data were combined as a single control group. All recordings were performed at room temperature (23°C) using an A-M 2400 amplifier (A-M Systems Inc., Carlsborg, WA, USA). Signals were low-pass filtered at 1 kHz and digitized at 5 kHz with a Digidata 1550A interface (Axon Instruments/Molecular Devices, Union City, CA, USA) and pCLAMP 10.0 software (Molecular Devices). Electrode capacitance was compensated after gigaohm (GΩ) seals were formed. The membrane capacitance, series resistance, and input resistance of the recorded ECs were measured by applying a +5 mV (100 ms) depolarizing voltage step from a holding potential of –60 mV. Cells with an input resistance <1 GΩ (smaller than 1 gigaohm) were discarded. The membrane capacitance reading was used as the value for whole cell capacitance (in pF). The outward currents (in pA) were elicited with a step-command from a holding potential at -60 mV to 40 mV for 300 ms. From the same cell, the total outward current containing K_Ca_3.1 was first recorded, followed by perfusion of 10 μM TRAM-34 (K_Ca_3.1 inhibitor) to the recording chamber for 5 minutes, and then a second current elicited and recorded in the presence of TRAM-34. The K_Ca_3.1 current from a single cell was isolated by a subtraction between the two recorded currents in the absence or presence of TRAM-34. The current density (pA/pF) was obtained by dividing the K_Ca_3.1 current amplitude (measured at 200 ms; the tau point) by the whole cell capacitance. The membrane potentials were recorded under the current-clamp mode by injecting a 20 pA current for 750 ms.

### MTT assays

The proliferation of HUVECs was determined using Tetrazoliumdye 3-(4,5-dimethylthiazol-2-yl)-2,5-diphenyltetrazolium bromide (MTT) assays (Thermo Fisher Scientific, Waltham, MA, USA) as we previously described [13, 14, 39]. HUVECs were seeded onto 24-well plates in EGM culture medium and allowed to adhere overnight. When cultures reached 60% confluency, peptide Lv (500 ng/ml), VEGF (5 ng/ml), DMH4 (5 μM), and TRAM-34 (10 μM) were added to cells and continuously incubated for another 48 hours. On the day of the assays, cells were incubated with the MTT solution (1.2 mM final concentration) for 4 hours at 37°C, after which the solution was removed, and 10% sodium dodecyl sulfate was added to break the plasma membrane. The absorbance at 560 nm was measured using a microplate reader (Awareness Technology Inc., Palm City, FL, USA).

### Immunoblot analysis

Cultured ECs were treated with PBS (vehicle control) or peptide Lv (500 ng/ml) for 4 hours then harvested and prepared for immunoblot analysis as we described previously [12, 13]. In brief, cells were lysed with a RIPA lysis buffer, and proteins were denatured by mixing with 2X Lamelli sample buffer and heating for 5 minutes at 95 ºC. Samples were separated using a 10% SDS-polyacrylamide gel and transferred to a nitrocellulose membrane. Membranes were incubated with the primary antibodies overnight at 4 ºC. The primary antibodies used were rabbit polyclonal anti-KCNN4, (K_Ca_3.1, #APC-0641:200; Alomone Labs, Jerusalem, Israel), rabbit polyclonal anti-KCNN3 (K_Ca_2.3, #APC-025; 1:200; Alomone Labs, Jerusalem, Israel), rabbit polyclonal anti-Kir6.1 (1:500; #NBP1-87710, Novus Biologicals, Littleton, CO, USA), and rabbit monoclonal β-actin (1:1000; #4970S, Cell Signaling Technologies, Danvers, MA, USA). Membranes were then washed with a TBS-tween solution followed by incubating with an anti-rabbit IgG HRP-linked antibody (1:1000; #7074S, Cell Signaling Technologies, Danvers, MA, USA) for 1 hour at room temperature (23 ºC). Bands were visualized using Super Signal West Pico or Femto chemiluminescent substrate (#34078 or #34096, Pierce Biotechnology Inc., Rockford, IL, USA). Membranes were scanned using an immunoblot scanner (LI-COR Biosciences, Lincoln, NE, USA). Band intensities were quantified using Image J (National Institutes of Health; NIH, Bethesda, MA, USA). The band intensities were first normalized to the internal control, β-actin, and subsequently, the relative changes were quantified according to the method described by Janes [40].

### Quantitative PCR (qPCR)

qPCR was performed as described previously [12–14]. After the cells were collected, total ribonucleic acid (RNA) from each sample was prepared by using a commercially available purification kit (RNeasy kit; #74106, Qiagen, Germantown, MD, USA). From each sample, 500 ng of total RNA was used to quantify messenger (mRNA) by qPCR using a High-Capacity cDNA Reverse Transcription Kit (#4368814, Applied Biosystems, Grand Island, NY, USA), Taqman qPCR master mix (#4444556; Applied Biosystems, Grand Island, NY, USA), and SYBR green supermix ROX (#95055–500, QuantaBio, Beverly, MA, USA) with a CFX Connect Real-Time PCR Detection System (Bio-Rad, Hercules, CA, USA). Primers used were purchased from Life Technologies (Carlsbad, CA, USA): *Kcnn4* (Forward: 5’-ATCTCCAAGATGCACATGATCC-3’; Reverse: 5’-TAGCCTGGTTCCTCCTCGTG-3’). *TRPV4* (Forward: 5’-CCAAGTACCCCGTGGTCTTCATC-3’; Reverse: 5’-AGGATGGTGGTGGCCCAC-3’); and *β-actin* (Forward: 5’-CAACGGCTCCGGCATGTGCAA-3’; Reverse 5’-GTACATGGCTGGGGTGTTGAAGGTCTC-3’).

For each experiment, a standard curve was generated with known quantities of RNAs loaded in serial dilutions (i.e., 2, 1, 1/2, 1/4, 1/8, 1/16, and 1/32). The cycle values, corresponding to the log values of the standard curve quantities, were used to generate a linear regression formula. The amplification efficiency of the qPCR reactions (90–100%) was calculated using the standard curve. The quantification of sample RNA was calculated by the 2^(-ΔΔCt)^ method [41] using *β-actin* as the internal control.

### Statistical analysis

All data are presented as mean ± standard error of the mean (SEM). Differences between two groups were analyzed using the student’s *t-*test. Differences between multiple groups were analyzed by one-way ANOVA and Tukey *post hoc* tests. The statistical software was Origin 8.6 (OriginLab, Northampton, MA, USA). Throughout, *p*<0.05 was considered significant.

## Results

### Peptide Lv hyperpolarizes the membrane potentials of ECs

**Fig 1:**
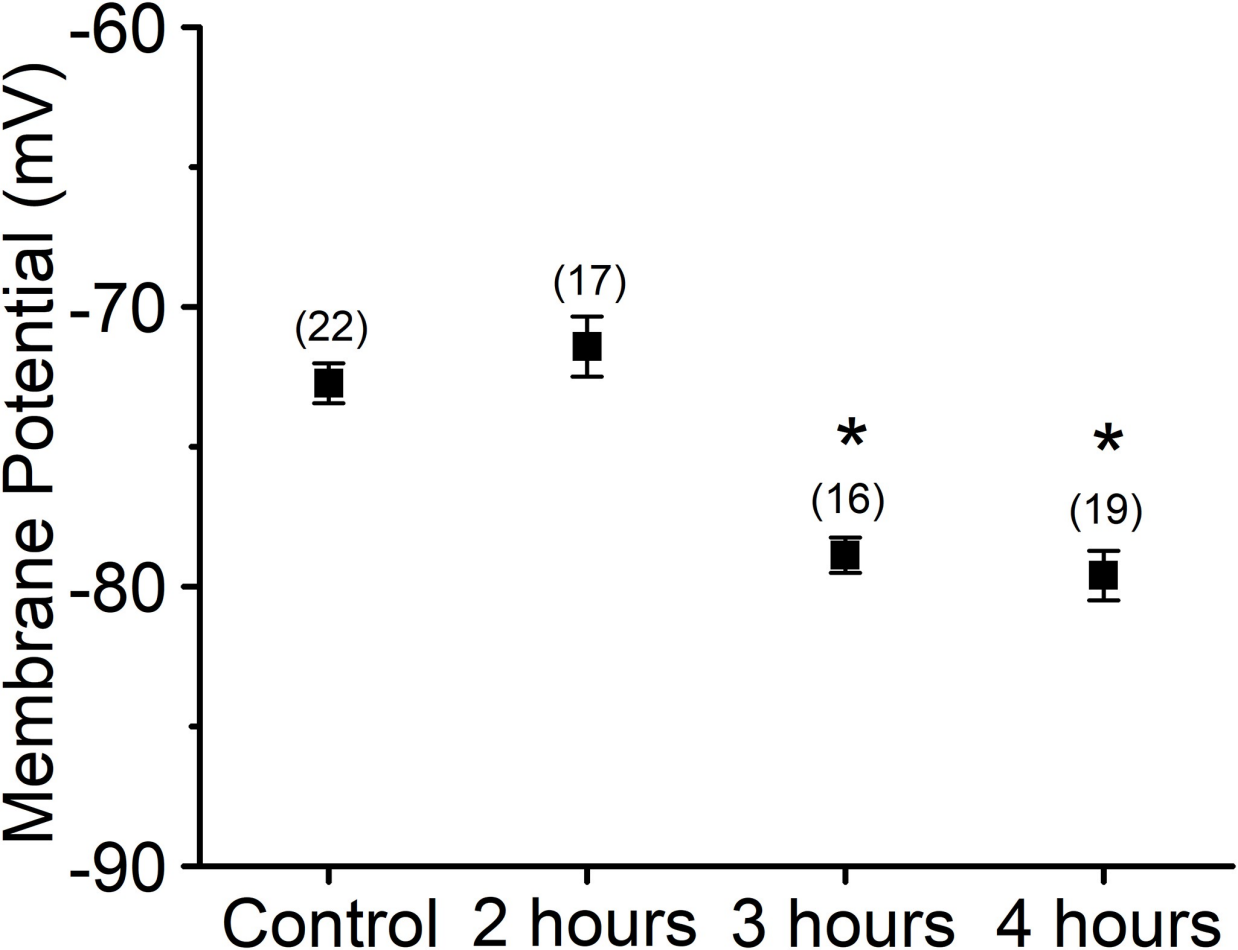
Peptide Lv hyperpolarizes the membrane potential of ECs. HUVECs were seeded onto glass coverslips and kept in an incubator for 48 hours to allow the cells to adhere. Cells were then treated with PBS (vehicle control) or peptide Lv (500 ng/ml) for 2, 3, or 4 hours prior to whole-cell current-clamp recordings. Membrane potentials were significantly hyperpolarized after treatments with peptide Lv (3 hr: -78.88 ± 0.63 mV and 4 hr: -79.61 ± 0.88 mV, respectively) compared to the PBS-treated controls (-72.73 ± 0.71 mV). Treatment with peptide Lv for 2 hours did not affect the EC membrane potential significantly (-71.42 ± 1.07 mV). One-way ANOVA followed with Tukey *post hoc* tests were used for statistical analyses; n = 16–19 for each group; *p<0.05.

We previously showed that peptide Lv promotes vasodilation and angiogenesis [14]. Membrane hyperpolarization in ECs is an essential step in EC-dependent vasodilation and angiogenesis [18, 24, 42, 43], so we first tested whether peptide Lv could elicit EC hyperpolarization. Using whole-cell current-clamp recordings, we found that treatment with peptide Lv (500 ng/ml) for 3 or 4 hours in cultures elicited membrane hyperpolarization of HUVECs compared to the vehicle control (PBS; Fig 1). The average membrane potential for HUVECs without peptide Lv is -72.73 ± 0.71 mV. After HUVECs were treated with peptide Lv for 3 or 4 hours, the average membrane potential was -78.88 ± 0.63 mV and -79.61 ± 0.88 mV, respectively. Thus, peptide Lv-elicited vasodilation and angiogenesis is in part through hyperpolarizing the EC plasma membrane.

### The intermediate-conductance calcium-dependent K^+^ channel (K_Ca_3.1) is a major player in peptide Lv-elicited endothelial hyperpolarization

**Fig 2:**
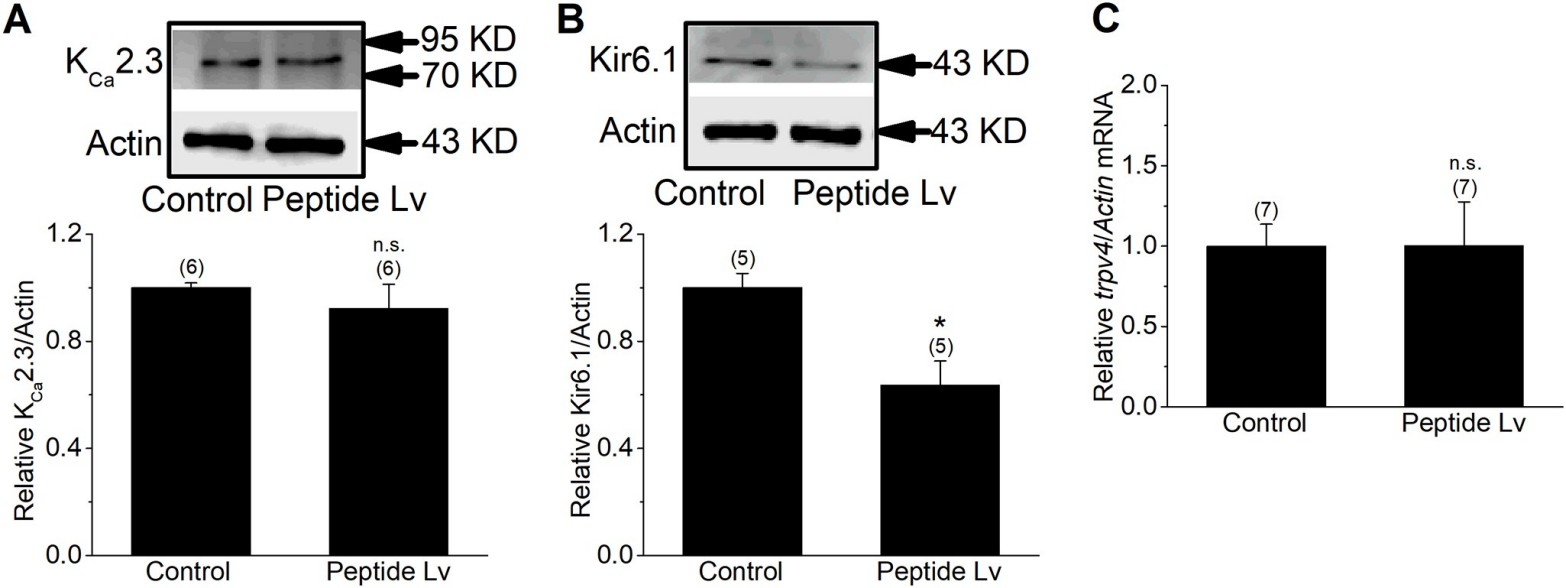
Peptide Lv does not increase the expressions of K_Ca_2.3, Kir6.1, or TRPV4 in ECs. Endothelial cells were treated with PBS (vehicle control) or peptide Lv (500 ng/ml) for 4 hours and processed for immunoblotting or qPCR. Peptide Lv did not increase (A) K_Ca_2.3, (B) Kir6.1, or (C) TRPV4 in cultured HUVECs. Student’s *t*-test was used for statistical analyses; *p<0.05.

**Fig 3:**
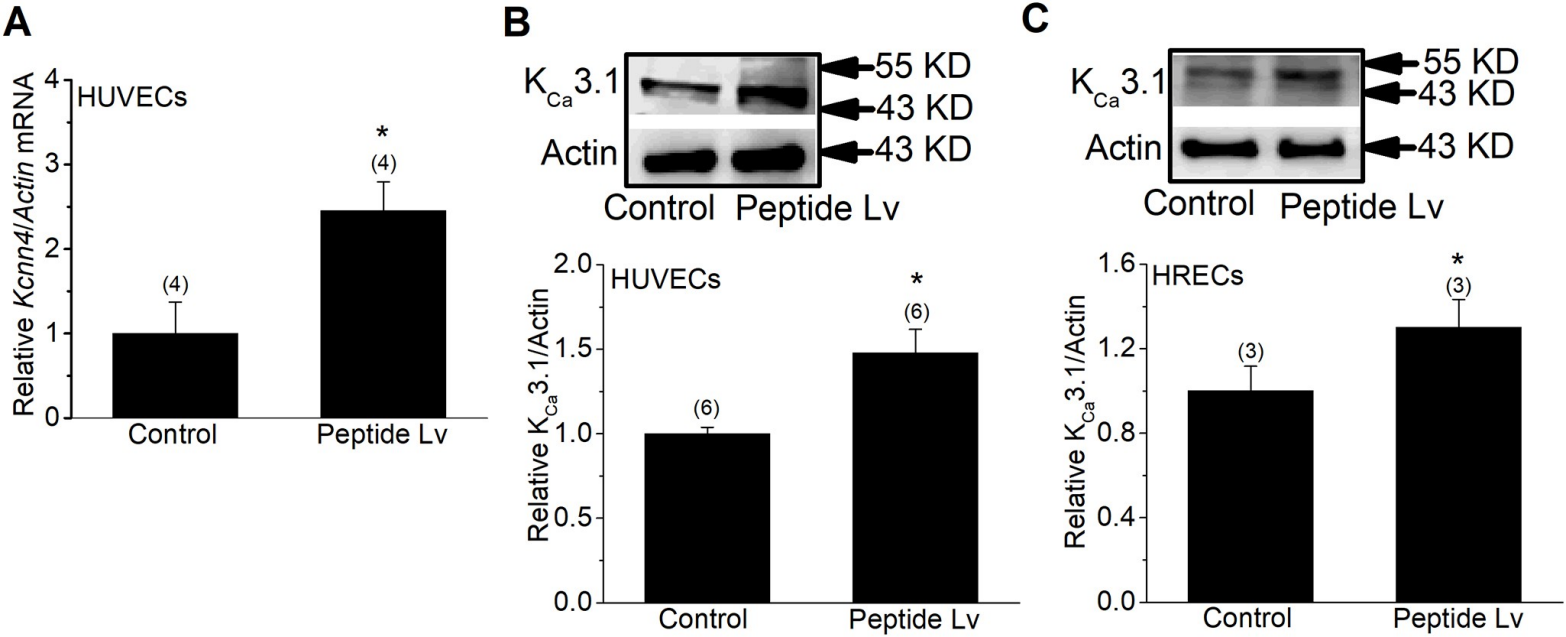
Peptide Lv increases the mRNA and protein levels of K_Ca_3.1 (*Kcnn4*) in ECs. Endothelial cells were treated with PBS (vehicle control) or peptide Lv (500 ng/ml) for 4 hours and processed for qPCR or immunoblotting. Peptide Lv increased the mRNA (A) and protein (B) levels of K_Ca_3.1 in cultured HUVECs. (C) Peptide Lv also increased the protein level of K_Ca_3.1 in HRECs. Student’s *t*-test was used for statistical analyses; *p<0.05.

Opening the endothelial K^+^ channels is required for EC hyperpolarization that leads to vasodilation [31, 44], so we next determined which K^+^ channels mediated peptide Lv-elicited EC hyperpolarization. Since peptide Lv elicited EC hyperpolarization after the cells were treated for 3 or 4 hours (Fig 1), we next tested which K^+^ channels were possibly upregulated by peptide Lv in cultured ECs. The HUVECs were first treated with peptide Lv (500 ng/ml) for 4 hours in cultures and subsequently harvested for qPCR or immunoblots to analyze various EC K^+^ channels that mediate EC hyperpolarization. Peptide Lv had no effect on the expression of the small-conductance calcium-dependent K^+^ channels (K_Ca_2.3; Fig 2A), and it decreased the protein level of ATP-sensitive K^+^ channels (Kir6.1; Fig 2B). Since decreased expression of Kir6.1 reduces endothelial hyperpolarization [30], and peptide Lv caused a decrease of Kir6.1 in cultured ECs, we eliminated Kir6.1 as a potential player in peptide Lv-mediated EC hyperpolarization. We previously showed that peptide Lv augments L-type voltage-gated calcium channels in cardiomyocytes and photoreceptors [12, 13], so it is possible that peptide Lv may augment other calcium channels in ECs, such as the transient receptor potential cation channel 4 (TRPV4) that indirectly contributes to EC hyperpolarization, as calcium influx through TRPV4 may allow calcium-dependent K^+^ channels to open [45, 46]. After cells were treated with peptide Lv, there was no change in the expression of TRPV4 (Fig 2C). However, we found that peptide Lv significantly increased the mRNA and protein expression of K_Ca_3.1 in HUVECs as well as in human retinal ECs (HRECs; Fig 3). These findings suggest that K_Ca_3.1 could be a key player in mediating endothelial hyperpolarization elicited by peptide Lv.

### Peptide Lv augments the K_Ca_3.1 current densities in ECs

**Fig 4:**
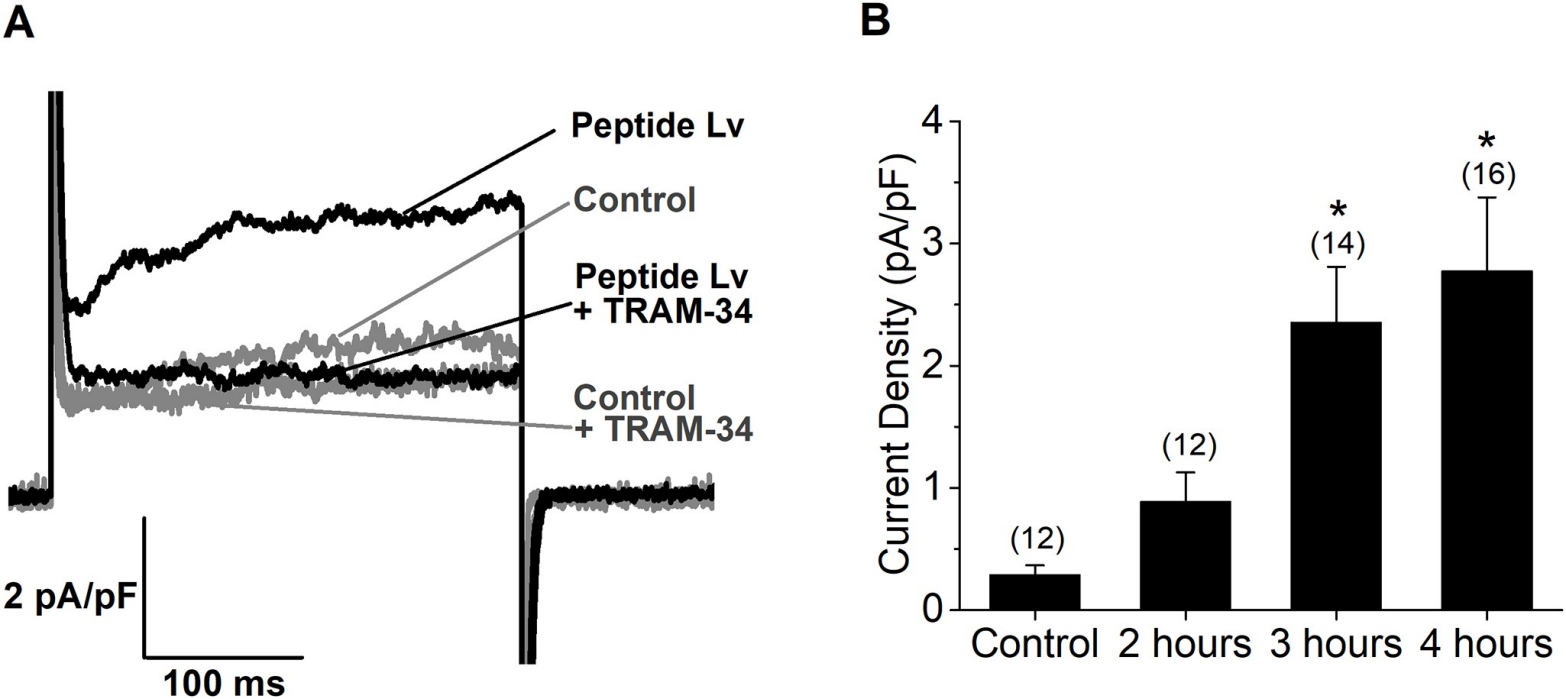
Peptide Lv augments the K_Ca_3.1 current densities in HUVECs. Whole-cell voltage-clamp recordings were performed on cultured HUVECs treated with PBS (vehicle control) or peptide Lv (500 ng/ml) for 2, 3, or 4 hours. The outward current was elicited with a step command from a holding potential at -60 mV to 40 mV for 300 ms. From the same cell, the first current was recorded in normal external solution, and then a second current was recorded in the presence of TRAM-34 (10 μM) to isolate the K_Ca_3.1 current. (A) Four representative traces recorded from two cells are shown. The two gray traces are recordings from the control cell (pretreated with PBS) in the absence (upper) or presence (lower) of TRAM-34. The two black traces are recordings from the cell pretreated with peptide Lv for 3 hours in the absence (upper) or presence (lower) of TRAM-34. (B) The current density (pA/pF) was obtained by dividing the K_Ca_3.1 current amplitude (measured at 200 ms; the tau point) by the whole cell capacitance. The K_Ca_3.1 current density was significantly larger in ECs treated with peptide Lv for 3 (2.36 ± 0.46 pA/pF) or 4 hours (2.77 ± 0.60 pA/pF) but not for 2 hours (0.89 ± 0.24 pA/pF), compared to the control treated with PBS (0.29 ± 0.08 pA/pF). One-way ANOVA followed with Tukey *post hoc* tests were used for statistical analyses; n = 12–16 for each group; *p<0.05.

As peptide Lv increased the mRNA and protein expression of K_Ca_3.1 in ECs, we next determined whether peptide Lv was able to increase the functional K_Ca_3.1. Cultured HUVECs were treated with PBS (vehicle control) or peptide Lv (500 ng/ml) for 2, 3, or 4 hours before the whole-cell voltage-clamp recordings of K_Ca_3.1 currents. TRAM-34 (10 μM; a K_Ca_3.1 inhibitor) was applied to the bath solution to isolate the K_Ca_3.1 current. HUVECs treated with peptide Lv (500 ng/ml) for 3 or 4 hours had significantly larger K_Ca_3.1 current densities compared to cells treated with a vehicle (Fig 4B). Thus, peptide Lv not only increased the mRNA and protein expression of K_Ca_3.1 but also augmented the K_Ca_3.1 activities in ECs. The augmentation of K_Ca_3.1 by peptide Lv positively correlated to peptide Lv-elicited EC hyperpolarization, indicating that K_Ca_3.1 was the K^+^ channel that mediated peptide Lv-elicited EC hyperpolarization.

### Peptide Lv promotes endothelial proliferation through K_Ca_3.1

**Fig 5:**
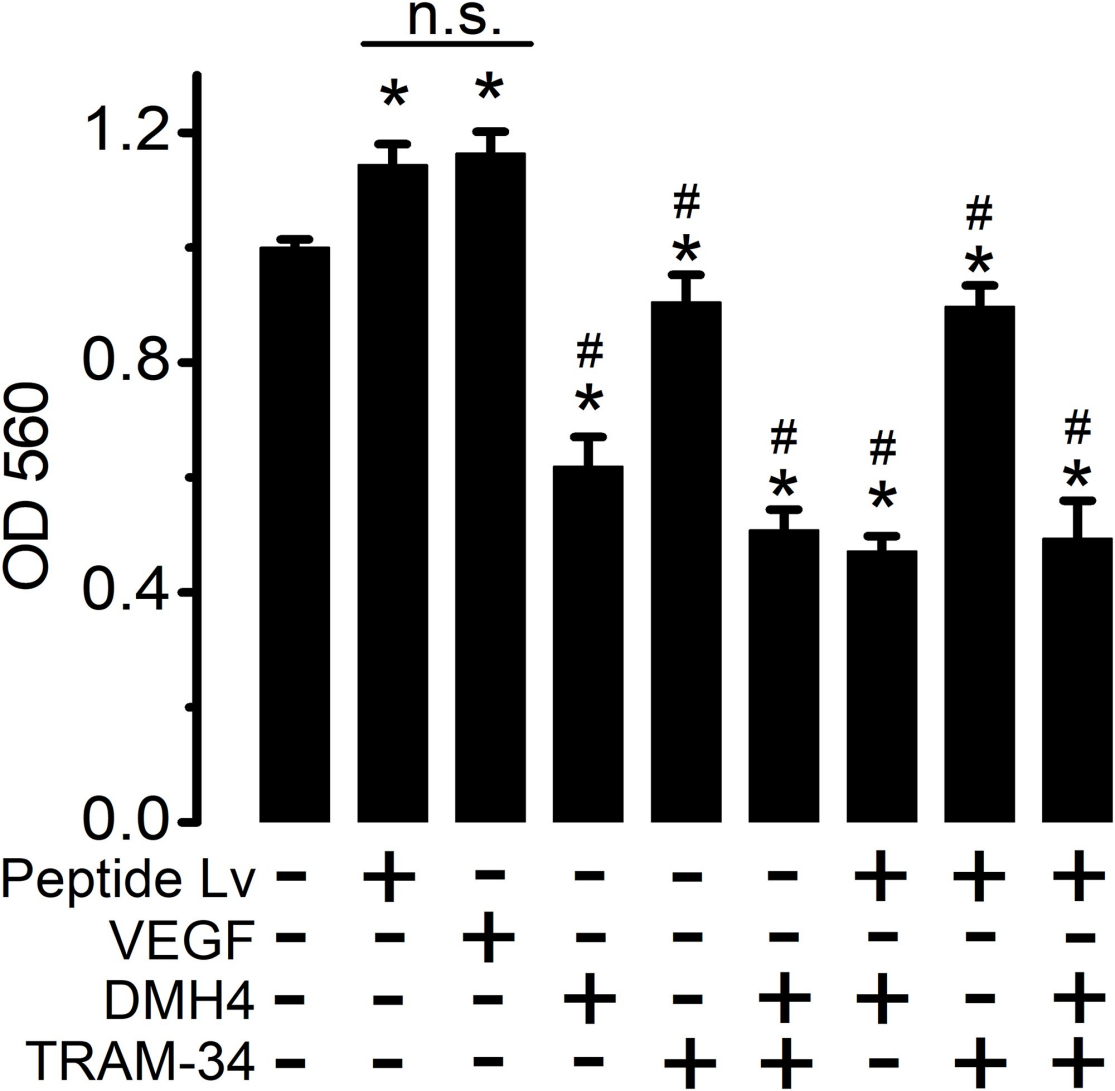
Inhibition of K_Ca_3.1 dampens peptide Lv-elicited EC proliferation. HUVECs were treated with peptide Lv (500 ng/ml), DMH4 (5 μM; a VEGFR2 inhibitor), TRAM-34 (10 μM, a K_Ca_3.1 inhibitor), or a combination of the above for 4 hours. Treatment with VEGF (5 ng/ml) served as a positive control and PBS as the vehicle control. The light absorbance was measured at 560 nm (OD 560 nm) for the MTT proliferation assays. One-way ANOVA followed with Tukey *post hoc* tests were used for statistical analyses; n = 12–15 for each group; “*” denotes a significant difference from the vehicle control; “#” denotes a significant difference from the peptide Lv treated group; *p*<0.05.

As we showed that peptide Lv facilitates angiogenesis in part through promoting EC proliferation [14], we tested whether blocking K_Ca_3.1 would inhibit peptide Lv-stimulated EC proliferation. Cultured HUVECs were treated with VEGF (5 ng/ml; positive control), peptide Lv (500 ng/ml), DMH4 (5 μM; VEGFR2 inhibitor), TRAM-34 (10 μM), or a combination for 4 hours. We previously showed that peptide Lv can bind to VEGFR2 and cause its activation through tyrosine phosphorylation [13], so it is not surprising that DMH4 reduced peptide Lv-elicited EC proliferation (Fig 5). Furthermore, blocking K_Ca_3.1 (with TRAM-34) or both VEGFR2 and K_Ca_3.1 (with DMH4+TRAM-34) significantly dampened peptide Lv-elicited EC proliferation. These data imply that peptide Lv-elicited angiogenesis is in part through K_Ca_3.1-dependent EC proliferation.

## Discussion

In this study, we investigated a potential mechanism in the promotion of angiogenesis by peptide Lv. Hyperpolarization of ECs leads to EC activation and angiogenesis [18, 21, 22]. We found that peptide Lv hyperpolarized ECs and increased the expression and current densities of K_Ca_3.1 after ECs were treated with peptide Lv for 3 hours in cultures. Peptide Lv-induced EC hyperpolarization was not through other K^+^ channels, since treatment with peptide Lv did not increase the expressions of K_ATP_, K_Ca_2.3, or TRPV4. Furthermore, blocking K_Ca_3.1 attenuated peptide Lv-stimulated EC proliferation. These results show that peptide Lv-elicited EC hyperpolarization was through the augmentation of K_Ca_3.1, and EC K_Ca_3.1 was involved in peptide Lv-elicited angiogenesis. While it might appear that changes of K_Ca_3.1 current density are more prominent than the changes of its protein expression, this is due to the different methods and data analyses. The protein expressions detected via Western blots are relative data (normalized with actin) from a culture dish with 80% confluency of ECs (for n = 1). The current density was obtained from the patch-clamp recording of a single EC (for n = 1), which is not a relative datum. Thus, peptide Lv-elicited increases in K_Ca_3.1 protein expression (detected by Western blots) might not be directly reflected onto the recorded current densities (detected with patch-clamp recordings) and vice versa.

Regulation of vasomotion is critical in maintaining systemic blood flow, oxygen delivery, and the health of vessels and capillaries [47–49]. Naturally, vasodilation is a mechanism to widen blood vessels and enhance blood flow to supply oxygen and nutrients to local tissues [47, 48, 50, 51]. However, chronic vasodilation of existing vessels causes increased vascular permeability in downstream capillaries [42, 43, 52], stimulates angiogenesis [53, 54], and promotes pathological neovascularization in various diseases [55–60]. Increased blood flow causes elevated shear stress in downstream small vessels and capillaries, which facilitates pericyte recruitment and microvascular sprouting and growth [58–61]. For example, vasodilation in both retinal arterioles and venules are associated with pathological neovascularization observed in proliferative diabetic retinopathy [58, 62–64]. Vasodilators such as VEGF increase the incidence of early age-related macular degeneration by 70% [57]. Retinal blood flow is increased in the proliferative phase of OIR and retinopathy of prematurity [65–68] and in the progression of choroidal neovascularization [69, 70]. Hence, chronic vasodilation is associated with the development of pathological neovascularization.

Previously, we showed that peptide Lv elicits vasodilation in coronary and retinal arterioles in a concentration-dependent manner [14]. VEGF-elicited vasodilation through its receptor (VEGFR2) is nitric oxide (NO)-dependent [15]. In contrast to VEGF, peptide Lv-elicited vasodilation is not completely attenuated by L-N^G^-Nitro arginine methyl ester (L-NAME), a NO synthase inhibitor [14], suggesting that peptide Lv has an NO-independent pathway that mediates vasodilation and possible angiogenesis. In the present study, we showed a new route of peptide Lv’s action. Peptide Lv-caused EC hyperpolarization through augmentation of K_Ca_3.1 can be an additional pathway in peptide Lv-elicited vasodilation and angiogenesis. Whether peptide Lv-elicited augmentation of K_Ca_3.1 in ECs mediates the NO-independent vasodilation will need to be further examined in the future.

The angiogenic property of peptide Lv is in part through binding to VEGFR2 [13], so the VEGFR2 antagonist DMH4 dampened peptide Lv-elicited EC proliferation. As peptide Lv also has VEGF/VEGFR2/NO-independent actions in vasodilation, it is possible that peptide Lv might contribute to the recurrent neovascularization and/or the resistance to anti-VEGF agents in patients. We demonstrated that peptide Lv is important in early photoreceptor development [12] and promotes cardiomyocyte function [13]. A recent study showed that peptide Lv plays a role in immune responses [71]. Macrophages treated with lipopolysaccharide (LPS) produce an increased inflammatory response that is dampened by treating the cells with peptide Lv [71]. In addition, bone-marrow derived macrophages isolated from mice with genetically knocked-out peptide Lv have a reduced inflammatory response compared to the macrophages isolated from wild-type mice [71]. As peptide Lv is a newly discovered small endogenous peptide that is expressed in multiple tissues and cell types, there may be more unknown functions and bioactivities of peptide Lv that are to be explored in the future.

## Supporting information

S1 Fig The original Western immunoblot images. The original Western immunoblot images included in Fig 2 is in S1 Fig.
(TIF)

S2 Fig The original Western immunoblot images. The original Western immunoblot images included in Fig 3 is in S2 Fig.
(TIF)

## References

[1] Yehya AHS, Asif M, Petersen SH, Subramaniam AV, Kono K, Majid A, et al. Angiogenesis: Managing the Culprits behind Tumorigenesis and Metastasis. Medicina (Kaunas). 2018;54(1):8 doi: 10.3390/medicina54010008 30344239 PMC6037250

[2] Sedding DG, Boyle EC, Demandt JAF, Sluimer JC, Dutzmann J, Haverich A, et al. Vasa Vasorum Angiogenesis: Key Player in the Initiation and Progression of Atherosclerosis and Potential Target for the Treatment of Cardiovascular Disease. Front Immunol. 2018;9:706 doi: 10.3389/fimmu.2018.00706 29719532 PMC5913371

[3] Elshabrawy HA, Chen Z, Volin MV, Ravella S, Virupannavar S, Shahrara S. The pathogenic role of angiogenesis in rheumatoid arthritis. Angiogenesis. 2015;18(4):433–48 doi: 10.1007/s10456-015-9477-2 26198292 PMC4879881

[4] Cai X, McGinnis JF. Diabetic Retinopathy: Animal Models, Therapies, and Perspectives. J Diabetes Res. 2016;2016:3789217 doi: 10.1155/2016/3789217 26881246 PMC4736804

[5] Carmeliet P. VEGF as a key mediator of angiogenesis in cancer. Oncology. 2005;69 Suppl 3:4–10 doi: 10.1159/000088478 16301830

[6] Campochiaro PA. Ocular neovascularization. J Mol Med (Berl). 2013;91(3):311–21 doi: 10.1007/s00109-013-0993-5 23329331 PMC3584193

[7] Aiello LP, Avery RL, Arrigg PG, Keyt BA, Jampel HD, Shah ST, et al. Vascular endothelial growth factor in ocular fluid of patients with diabetic retinopathy and other retinal disorders. N Engl J Med. 1994;331(22):1480–7 doi: 10.1056/NEJM199412013312203 7526212

[8] Pe’er J, Folberg R, Itin A, Gnessin H, Hemo I, Keshet E. Upregulated expression of vascular endothelial growth factor in proliferative diabetic retinopathy. Br J Ophthalmol. 1996;80(3):241–5 doi: 10.1136/bjo.80.3.241 8703862 PMC505435

[9] Lux A, Llacer H, Heussen FM, Joussen AM. Non-responders to bevacizumab (Avastin) therapy of choroidal neovascular lesions. Br J Ophthalmol. 2007;91(10):1318–22 doi: 10.1136/bjo.2006.113902 17537784 PMC2000982

[10] Tranos P, Vacalis A, Asteriadis S, Koukoula S, Vachtsevanos A, Perganta G, et al. Resistance to antivascular endothelial growth factor treatment in age-related macular degeneration. Drug Des Devel Ther. 2013;7:485–90 doi: 10.2147/DDDT.S43470 23818759 PMC3692343

[11] Binder S. Loss of reactivity in intravitreal anti-VEGF therapy: tachyphylaxis or tolerance? Br J Ophthalmol. 2012;96(1):1–2 doi: 10.1136/bjophthalmol-2011-301236 22157632

[12] Shi L, Ko ML, Abbott LC, Ko GY. Identification of Peptide lv, a novel putative neuropeptide that regulates the expression of L-type voltage-gated calcium channels in photoreceptors. PLoS One. 2012;7(8):e43091 doi: 10.1371/journal.pone.0043091 22912796 PMC3418253

[13] Shi L, Ko S, Ko ML, Kim AJ, Ko GY. Peptide Lv augments L-type voltage-gated calcium channels through vascular endothelial growth factor receptor 2 (VEGFR2) signaling. Biochim Biophys Acta. 2015;1853(5):1154–64 doi: 10.1016/j.bbamcr.2015.02.007 25698653 PMC4382007

[14] Shi L, Zhao M, Abbey CA, Tsai SH, Xie W, Pham D, et al. Newly Identified Peptide, Peptide Lv, Promotes Pathological Angiogenesis. J Am Heart Assoc. 2019;8(22):e013673 doi: 10.1161/JAHA.119.013673 31698979 PMC6915261

[15] Hein TW, Rosa RH Jr., Ren Y, Xu W, Kuo L. VEGF Receptor-2-Linked PI3K/Calpain/SIRT1 Activation Mediates Retinal Arteriolar Dilations to VEGF and Shear Stress. Invest Ophthalmol Vis Sci. 2015;56(9):5381–9 doi: 10.1167/iovs15-16950 26284543 PMC4544352

[16] Hammes HP. Diabetic retinopathy: hyperglycaemia, oxidative stress and beyond. Diabetologia. 2018;61(1):29–38 doi: 10.1007/s00125-017-4435-8 28942458

[17] Hammes HP, Lin J, Bretzel RG, Brownlee M, Breier G. Upregulation of the vascular endothelial growth factor/vascular endothelial growth factor receptor system in experimental background diabetic retinopathy of the rat. Diabetes. 1998;47(3):401–6 doi: 10.2337/diabetes.47.3.401 9519746

[18] Dabisch PA, Liles JT, Taylor JT, Sears BW, Saenz R, Kadowitz PJ. Role of potassium channels in the nitric oxide-independent vasodilator response to acetylcholine. Pharmacol Res. 2004;49(3):207–15 doi: 10.1016/j.phrs.2003.09.010 14726215

[19] Hein TW, Yuan Z, Rosa RH Jr., Kuo L. Requisite roles of A2A receptors, nitric oxide, and KATP channels in retinal arteriolar dilation in response to adenosine. Invest Ophthalmol Vis Sci. 2005;46(6):2113–9 doi: 10.1167/iovs.04-1438 15914631

[20] Michaelis UR, Fleming I. From endothelium-derived hyperpolarizing factor (EDHF) to angiogenesis: Epoxyeicosatrienoic acids (EETs) and cell signaling. Pharmacol Ther. 2006;111(3):584–95 doi: 10.1016/j.pharmthera.2005.11.003 16380164

[21] Adams RH, Alitalo K. Molecular regulation of angiogenesis and lymphangiogenesis. Nat Rev Mol Cell Biol. 2007;8(6):464–78 doi: 10.1038/nrm2183 17522591

[22] Shepherd JT, Katusic ZS. Endothelium-derived vasoactive factors: I. Endothelium-dependent relaxation. Hypertension. 1991;18(5 Suppl):III76–85 doi: 10.1161/01.hyp.18.5_suppl.iii76 1937690

[23] Edwards G, Feletou M, Weston AH. Endothelium-derived hyperpolarising factors and associated pathways: a synopsis. Pflugers Arch. 2010;459(6):863–79 doi: 10.1007/s00424-010-0817-1 20383718

[24] Edwards G, Dora KA, Gardener MJ, Garland CJ, Weston AH. K+ is an endothelium-derived hyperpolarizing factor in rat arteries. Nature. 1998;396(6708):269–72 doi: 10.1038/24388 9834033

[25] Grgic I, Kaistha BP, Hoyer J, Kohler R. Endothelial Ca+-activated K+ channels in normal and impaired EDHF-dilator responses—relevance to cardiovascular pathologies and drug discovery. Br J Pharmacol. 2009;157(4):509–26 doi: 10.1111/j.1476-5381.2009.00132.x 19302590 PMC2707963

[26] Grgic I, Eichler I, Heinau P, Si H, Brakemeier S, Hoyer J, et al. Selective blockade of the intermediate-conductance Ca2+-activated K+ channel suppresses proliferation of microvascular and macrovascular endothelial cells and angiogenesis in vivo. Arterioscler Thromb Vasc Biol. 2005;25(4):704–9 doi: 10.1161/01.ATV.0000156399.12787.5c 15662023

[27] Yang H, Li X, Ma J, Lv X, Zhao S, Lang W, et al. Blockade of the intermediate-conductance Ca(2+)-activated K+ channel inhibits the angiogenesis induced by epidermal growth factor in the treatment of corneal alkali burn. Exp Eye Res. 2013;110:76–87 doi: 10.1016/j.exer.2013.02.015 23482085

[28] Ledoux J, Werner ME, Brayden JE, Nelson MT. Calcium-activated potassium channels and the regulation of vascular tone. Physiology (Bethesda). 2006;21:69–78 doi: 10.1152/physiol.00040.2005 16443824

[29] Aziz Q, Thomas AM, Gomes J, Ang R, Sones WR, Li Y, et al. The ATP-sensitive potassium channel subunit, Kir6.1, in vascular smooth muscle plays a major role in blood pressure control. Hypertension. 2014;64(3):523–9 doi: 10.1161/HYPERTENSIONAHA.114.03116 24914196

[30] Aziz Q, Li Y, Anderson N, Ojake L, Tsisanova E, Tinker A. Molecular and functional characterization of the endothelial ATP-sensitive potassium channel. J Biol Chem. 2017;292(43):17587–97 doi: 10.1074/jbc.M117.810325 28893911 PMC5663864

[31] Hein TW, Xu W, Kuo L. Dilation of retinal arterioles in response to lactate: role of nitric oxide, guanylyl cyclase, and ATP-sensitive potassium channels. Invest Ophthalmol Vis Sci. 2006;47(2):693–9 doi: 10.1167/iovs.05-1224 16431969

[32] Fan JS, Palade P. Perforated patch recording with beta-escin. Pflugers Arch. 1998;436(6):1021–3 doi: 10.1007/pl00008086 9799421

[33] Huang CC, Ko ML, Ko GY. A new functional role for mechanistic/mammalian target of rapamycin complex 1 (mTORC1) in the circadian regulation of L-type voltage-gated calcium channels in avian cone photoreceptors. PLoS One. 2013;8(8):e73315 doi: 10.1371/journal.pone.0073315 23977383 PMC3747127

[34] Ko GY, Shi L, Ko ML. Circadian regulation of ion channels and their functions. J Neurochem. 2009;110(4):1150–69 doi: 10.1111/j.1471-4159.2009.06223.x 19549279 PMC2819050

[35] Li W, Halling DB, Hall AW, Aldrich RW. EF hands at the N-lobe of calmodulin are required for both SK channel gating and stable SK-calmodulin interaction. J Gen Physiol. 2009;134(4):281–93 doi: 10.1085/jgp.200910295 19752189 PMC2757765

[36] Jenkins DP, Yu W, Brown BM, Lojkner LD, Wulff H. Development of a QPatch automated electrophysiology assay for identifying KCa3.1 inhibitors and activators. Assay Drug Dev Technol. 2013;11(9–10):551–60 doi: 10.1089/adt.2013.543 24351043 PMC3870577

[37] Papassotiriou J, Kohler R, Prenen J, Krause H, Akbar M, Eggermont J, et al. Endothelial K(+) channel lacks the Ca(2+) sensitivity-regulating beta subunit. FASEB J. 2000;14(7):885–94. 10783142

[38] Schoenmakers TJ, Visser GJ, Flik G, Theuvenet AP. CHELATOR: an improved method for computing metal ion concentrations in physiological solutions. Biotechniques. 1992;12(6):870–4, 6–9. 1642895

[39] Mosmann T. Rapid colorimetric assay for cellular growth and survival: application to proliferation and cytotoxicity assays. J Immunol Methods. 1983;65(1–2):55–63 doi: 10.1016/0022-1759(83)90303-4 6606682

[40] Janes KA. An analysis of critical factors for quantitative immunoblotting. Sci Signal. 2015;8(371):rs2 doi: 10.1126/scisignal.2005966 25852189 PMC4401487

[41] Livak KJ, Schmittgen TD. Analysis of relative gene expression data using real-time quantitative PCR and the 2(-Delta Delta C(T)) Method. Methods. 2001;25(4):402–8 doi: 10.1006/meth.2001.1262 11846609

[42] Hood JD, Meininger CJ, Ziche M, Granger HJ. VEGF upregulates ecNOS message, protein, and NO production in human endothelial cells. Am J Physiol. 1998;274(3):H1054–8 doi: 10.1152/ajpheart.1998.274.3.H1054 9530221

[43] Conway EM, Collen D, Carmeliet P. Molecular mechanisms of blood vessel growth. Cardiovasc Res. 2001;49(3):507–21 doi: 10.1016/s0008-6363(00)00281-9 11166264

[44] Nilius B, Droogmans G. Ion channels and their functional role in vascular endothelium. Physiol Rev. 2001;81(4):1415–59 doi: 10.1152/physrev.2001.81.4.1415 11581493

[45] Seki T, Goto K, Kiyohara K, Kansui Y, Murakami N, Haga Y, et al. Downregulation of Endothelial Transient Receptor Potential Vanilloid Type 4 Channel and Small-Conductance of Ca2+-Activated K+ Channels Underpins Impaired Endothelium-Dependent Hyperpolarization in Hypertension. Hypertension. 2017;69(1):143–53 doi: 10.1161/HYPERTENSIONAHA.116.07110 27872234

[46] Earley S, Pauyo T, Drapp R, Tavares MJ, Liedtke W, Brayden JE. TRPV4-dependent dilation of peripheral resistance arteries influences arterial pressure. Am J Physiol Heart Circ Physiol. 2009;297(3):H1096–102 doi: 10.1152/ajpheart.00241.2009 19617407 PMC2755979

[47] Sato A, Terata K, Miura H, Toyama K, Loberiza FR Jr., Hatoum OA, et al. Mechanism of vasodilation to adenosine in coronary arterioles from patients with heart disease. Am J Physiol Heart Circ Physiol. 2005;288(4):H1633–40 doi: 10.1152/ajpheart.00575.2004 15772334

[48] Costa F, Biaggioni I. Role of nitric oxide in adenosine-induced vasodilation in humans. Hypertension. 1998;31(5):1061–4 doi: 10.1161/01.hyp.31.5.1061 9576114

[49] Clifford PS. Local control of blood flow. Adv Physiol Educ. 2011;35(1):5–15 doi: 10.1152/advan.00074.2010 21385995

[50] Krogh A. The number and distribution of capillaries in muscles with calculations of the oxygen pressure head necessary for supplying the tissue. J Physiol. 1919;52(6):409–15 doi: 10.1113/jphysiol.1919.sp001839 16993405 PMC1402716

[51] Krogh A. The supply of oxygen to the tissues and the regulation of the capillary circulation. J Physiol. 1919;52(6):457–74 doi: 10.1113/jphysiol.1919.sp001844 16993410 PMC1402718

[52] Ashina K, Tsubosaka Y, Kobayashi K, Omori K, Murata T. VEGF-induced blood flow increase causes vascular hyper-permeability in vivo. Biochem Biophys Res Commun. 2015;464(2):590–5 doi: 10.1016/j.bbrc.2015.07.014 26163262

[53] Crawford Y, Kasman I, Yu L, Zhong C, Wu X, Modrusan Z, et al. PDGF-C mediates the angiogenic and tumorigenic properties of fibroblasts associated with tumors refractory to anti-VEGF treatment. Cancer Cell. 2009;15(1):21–34 doi: 10.1016/j.ccr.2008.12.004 19111878

[54] Pili R, Chang J, Partis RA, Mueller RA, Chrest FJ, Passaniti A. The alpha-glucosidase I inhibitor castanospermine alters endothelial cell glycosylation, prevents angiogenesis, and inhibits tumor growth. Cancer Res. 1995;55(13):2920–6. 7540952

[55] Takahashi H, Shibuya M. The vascular endothelial growth factor (VEGF)/VEGF receptor system and its role under physiological and pathological conditions. Clin Sci (Lond). 2005;109(3):227–41 doi: 10.1042/CS20040370 16104843

[56] Tugues S, Koch S, Gualandi L, Li X, Claesson-Welsh L. Vascular endothelial growth factors and receptors: anti-angiogenic therapy in the treatment of cancer. Mol Aspects Med. 2011;32(2):88–111 doi: 10.1016/j.mam.2011.04.004 21565214

[57] Klein R, Myers CE, Klein BE. Vasodilators, blood pressure-lowering medications, and age-related macular degeneration: the Beaver Dam Eye Study. Ophthalmology. 2014;121(8):1604–11 doi: 10.1016/j.ophtha.2014.03.005 24793737 PMC4122609

[58] Stefansson E, Landers MB 3rd, Wolbarsht ML. Oxygenation and vasodilatation in relation to diabetic and other proliferative retinopathies. Ophthalmic Surg. 1983;14(3):209–26. 6190118

[59] Kolluru GK, Sinha S, Majumder S, Muley A, Siamwala JH, Gupta R, et al. Shear stress promotes nitric oxide production in endothelial cells by sub-cellular delocalization of eNOS: A basis for shear stress mediated angiogenesis. Nitric Oxide. 2010;22(4):304–15 doi: 10.1016/j.niox.2010.02.004 20188204

[60] Galie PA, Nguyen DH, Choi CK, Cohen DM, Janmey PA, Chen CS. Fluid shear stress threshold regulates angiogenic sprouting. Proc Natl Acad Sci U S A. 2014;111(22):7968–73 doi: 10.1073/pnas.1310842111 24843171 PMC4050561

[61] Uchida C, Nwadozi E, Hasanee A, Olenich S, Olfert IM, Haas TL. Muscle-derived vascular endothelial growth factor regulates microvascular remodelling in response to increased shear stress in mice. Acta Physiol (Oxf). 2015;214(3):349–60 doi: 10.1111/apha.12463 25659833

[62] da Silva AV, Gouvea SA, da Silva AP, Bortolon S, Rodrigues AN, Abreu GR, et al. Changes in retinal microvascular diameter in patients with diabetes. Int J Gen Med. 2015;8:267–73 doi: 10.2147/IJGM.S83749 26345217 PMC4554448

[63] Lecleire-Collet A, Audo I, Aout M, Girmens JF, Sofroni R, Erginay A, et al. Evaluation of retinal function and flicker light-induced retinal vascular response in normotensive patients with diabetes without retinopathy. Invest Ophthalmol Vis Sci. 2011;52(6):2861–7 doi: 10.1167/iovs.10-5960 21282578

[64] Nguyen TT, Kawasaki R, Wang JJ, Kreis AJ, Shaw J, Vilser W, et al. Flicker light-induced retinal vasodilation in diabetes and diabetic retinopathy. Diabetes Care. 2009;32(11):2075–80 doi: 10.2337/dc09-0075 19641162 PMC2768208

[65] Hartenstein S, Muller B, Metze B, Czernik C, Buhrer C. Blood flow assessed by color Doppler imaging in retinopathy of prematurity. J Perinatol. 2015;35(9):745–7 doi: 10.1038/jp.2015.45 25950917

[66] Matsumoto T, Itokawa T, Shiba T, Tomita M, Hine K, Mizukaki N, et al. Decreased ocular blood flow after photocoagulation therapy in neonatal retinopathy of prematurity. Jpn J Ophthalmol. 2017;61(6):484–93 doi: 10.1007/s10384-017-0536-7 28932922

[67] Matsumoto T, Saito Y, Itokawa T, Shiba T, Oba MS, Takahashi H, et al. Retinal VEGF levels correlate with ocular circulation measured by a laser speckle-micro system in an oxygen-induced retinopathy rat model. Graefes Arch Clin Exp Ophthalmol. 2017;255(10):1981–90 doi: 10.1007/s00417-017-3756-0 28791491

[68] Matsumoto T, Itokawa T, Shiba T, Tomita M, Hine K, Mizukaki N, et al. Intravitreal bevacizumab treatment reduces ocular blood flow in retinopathy of prematurity: a four-case report. Graefes Arch Clin Exp Ophthalmol. 2018;256(11):2241–7 doi: 10.1007/s00417-018-4063-0 29980917

[69] Rebhun CB, Moult EM, Ploner SB, Neto CM, Alibhai AY, Schottenhamml J, et al. Analyzing relative blood flow speeds in choroidal neovascularization using variable interscan time analysis OCT angiography. Ophthalmol Retina. 2018;2(4):306–19 doi: 10.1016/j.oret.2017.08.013 31047240 PMC6532791

[70] Spaide RF. Optical coherence tomography angiography signs of vascular abnormalization with antiangiogenic therapy for choroidal neovascularization. Am J Ophthalmol. 2015;160(1):6–16 doi: 10.1016/j.ajo.2015.04.012 25887628

[71] Mukai M, Uchida K, Okubo T, Takano S, Matsumoto T, Satoh M, et al. Regulation of Tumor Necrosis Factor-alpha by Peptide Lv in Bone Marrow Macrophages and Synovium. Front Med (Lausanne). 2021;8:702126 doi: 10.3389/fmed.2021.702126 34386509 PMC8353113

